# Metagenomic and culture-dependent characterization of between-field variation in phosphorus-cycling microbial communities and functional potential across tobacco–safflower rotation fields

**DOI:** 10.3389/fmicb.2026.1983930

**Published:** 2026-10-08

**Authors:** You Luo, Yu Wang, Zhufeng Shi, Zilin Li, Te Pu, Yanni Wang, Ming Ni, Xun He, Jing Li, Chenying Li, Xuewei Li, Peiwen Yang

**Affiliations:** 1 Lijiang Branch of Yunnan Provincial Tobacco Company, Lijiang, Yunnan, China; 2 Institute of Agricultural Environment and Resources, Yunnan Academy of Agricultural Sciences, Kunming, China

**Keywords:** metagenomics, phosphate-solubilizing bacteria, phosphorus cycling, rhizosphere soil, tobacco–safflower rotation

## Abstract

**Introduction:**

Rhizosphere microorganisms contribute to soil phosphorus (P) transformation and plant P acquisition, but P-cycling microbial communities and functional associations across tobacco–safflower rotation fields remain poorly characterized.

**Methods:**

Twenty-one safflower rhizosphere soil samples were collected from seven tobacco–safflower rotation fields in Lijiang, China. Soil physicochemical properties were measured, and shotgun metagenomics was used to characterize P-cycling bacterial communities and genes associated with P solubilization, mineralization, regulation, and transport. Culturable phosphate-solubilizing bacteria were isolated, preliminarily assigned taxonomically using 16S rRNA gene sequences, and evaluated by plate and liquid-culture assays.

**Results:**

Soil properties and P-cycling bacterial communities showed pronounced between-field variation. Metagenomic screening identified 6,019 non-redundant gene sequences assigned to 38 predefined P-cycling KO terms, with 460 genus-level bacterial taxa. Among 124 taxa shared across all fields, *Nocardioides, Arthrobacter, Streptomyces, Gaiella*, and *Marmoricola* were major dominant genera. PCoA and PERMANOVA showed significant field differences at the phylum (*R*^2^ = 0.983, *p* = 0.001) and genus (*R*^2^ = 0.964, *p* = 0.001) levels. Soil pH was associated with all four P-cycling functional modules. Exploratory random-forest analysis identified *Sphingomonas, Streptomyces, Nocardioides*, and *Arthrobacter* as candidate indicator taxa associated with variation in soil available P. Eighty-six culturable phosphate-solubilizing isolates representing 45 putative species-level taxa were obtained, with *Streptomyces* the most abundant genus (22.1%). *Streptomyces* isolates had solubilization index values of 1.10–1.89. The isolate with the highest SI, *Streptomyces* sp. LG-4, provisionally affiliated with *S. endophyticus*, reached a maximum net phosphate solubilization of 354.60 ± 7.22 mg L^−1^ on day 3.

**Conclusion:**

Soil physicochemical heterogeneity, particularly pH, was associated with between-field differences in P-cycling microbial communities and metagenomically inferred functional potential. Because no non-rotation or monoculture controls were included, these patterns should be interpreted as field-associated variation rather than effects of the rotation system itself. Both metagenomic and culture-dependent analyses highlighted *Streptomyces* as a candidate group of interest, while *Streptomyces* sp. LG-4 showed phosphate-solubilizing capacity under laboratory conditions and requires further taxonomic and functional validation.

## 1 Introduction

Phosphorus (P) is an essential macronutrient for plant growth and development and participates in key physiological processes, including energy metabolism, nucleic acid synthesis, and photosynthesis (Kafle et al., 2019; Pan et al., 2022). Although total P concentrations in soil are generally high, most soil P occurs as sparingly soluble inorganic P or organically bound forms, and only a small proportion is directly available for plant uptake. Tobacco is an important cash crop whose yield and quality are highly sensitive to soil nutrient supply. Although long-term heavy application of P fertilizers can meet crop nutrient demand, it may reduce P-use efficiency, disrupt soil nutrient balance, and increase agricultural non-point-source pollution (Liu J. Y. et al., 2023; Liu et al., 2017; Guelfi et al., 2022). Therefore, mobilizing poorly available soil P and improving P-use efficiency are important challenges for nutrient management and sustainable production in tobacco-growing soils.

Appropriate crop rotation can alter crop nutrient uptake, root exudation, and residue inputs, thereby modifying soil nutrient status and microbial communities and ultimately influencing soil P availability (Łukowiak et al., 2016; Duan et al., 2024). Previous studies have shown that rotation or intercropping of tobacco with maize, purple gromwell, and other crops can increase soil available P and other readily available nutrients to varying degrees (Sun et al., 2026; Zhong et al., 2024; Zhao et al., 2026). Rhizosphere niches created by different crops can also selectively enrich potential phosphate-solubilizing bacteria, arbuscular mycorrhizal fungi, and other microorganisms involved in nutrient transformation, while influencing soil P availability through changes in root organic acid exudation and P-fraction transformation (Sun et al., 2022; Liu X. et al., 2025; Yang et al., 2025; Liu Q. et al., 2025). In addition, crop rotation can alter the abundances of P-cycling functional genes such as *phoD, phnA*, and *phnP* and their associated microbial taxa (Wang et al., 2024; Xie et al., 2020). Collectively, these studies indicate that soil P transformation in crop-rotation systems is closely associated with rhizosphere microbial communities and their P-cycling functional potential, although responses vary substantially among crop combinations and regions.

Metagenomics enables characterization of the taxonomic composition and genetic functional potential of microbial communities associated with soil P cycling and can identify genes potentially involved in inorganic P mobilization, organic P mineralization, P transport, and P-responsive regulation through functional annotation (Wani et al., 2025; Wu et al., 2022). However, the presence and abundance of these genes reflect functional potential rather than gene expression or actual rates of P transformation. In addition, metagenomic analysis does not directly provide viable strains for further functional validation and application. In contrast, culture-dependent isolation of phosphate-solubilizing bacteria yields live microbial resources with observable phosphate-solubilizing phenotypes. Therefore, integrating metagenomic analysis with culture-dependent isolation can simultaneously characterize the taxonomic composition and functional potential of P-cycling microorganisms while enabling the recovery of culturable phosphate-solubilizing bacterial resources for further functional evaluation (Liang et al., 2020).

Safflower is an important specialty cash crop in Yunnan Province, and the rhizosphere environment of tobacco–safflower rotation systems may influence soil P transformation and related microbial processes. Nevertheless, the P-cycling bacterial taxa and functional genes present in different tobacco–safflower rotation fields, and their relationships with soil environmental factors, remain poorly characterized, particularly with respect to between-field variation. Accordingly, this study investigated safflower rhizosphere soils from seven tobacco–safflower rotation fields in Lijiang, Yunnan Province, China. We compared soil physicochemical properties among fields, characterized between-field variation in P-cycling bacterial taxa and functional genes, and identified environmental factors and potentially important bacterial taxa closely associated with P-cycling functions and variation in soil available P. In parallel, culturable phosphate-solubilizing bacteria were isolated and screened to identify strains with actual phosphate-solubilizing potential. This study aimed to characterize between-field variation in P-cycling microbial communities and their functional potential across tobacco–safflower rotation fields and to provide a theoretical basis for P management in tobacco-growing soils and the development of phosphate-solubilizing microbial resources.

## 2 Materials and methods

### 2.1 Study area and soil sampling

The study area was located in the Jiangbian dry-hot valley of Yongsheng County, Lijiang City, Yunnan Province, China, and included seven representative tobacco–safflower rotation fields in Qina Town (Banping, Liuguan, and Xilang villages), Sanchuan Town (Wenxiang, Xingwen, and Yangwu villages), and Liude Township (Liude Village). The study area has a mean annual temperature of 21.4 °C, mean annual precipitation of 868.2 mm, and annual sunshine duration of 2,421.4 h. Information on the sampling sites is provided in Supplementary Table S1.

Sampling was conducted in April 2025 at safflower harvest. The tobacco cultivar used in the rotation system was Yunyan 87, and the safflower cultivar was Dianhonghua 6. Three spatially independent sampling sites were established within each field and treated as biological replicates. At each replicate site, soil was collected from the 0–20 cm layer using a five-point composite sampling method with a soil auger sterilized with 75% ethanol. Loosely adhering non-rhizosphere soil was removed by gently shaking the roots, and rhizosphere soil tightly adhering within 0–5 mm of the root surface was collected. Stones, grass roots, crop residues, and other debris were removed. The five rhizosphere soil subsamples collected within each replicate site were combined and thoroughly homogenized to form one composite rhizosphere soil sample. Each composite rhizosphere soil sample constituted one experimental unit. Thus, three independent biological replicate samples were obtained from each field, yielding a total of 21 safflower rhizosphere soil samples. Each biological replicate was processed independently for soil physicochemical analysis, metagenomic sequencing, and culture-dependent isolation.

Each soil sample was divided into three subsamples. The first subsample was air-dried, ground, sieved, and stored in a desiccator for determination of soil physicochemical properties. The second subsample consisted of fresh soil placed in sterile centrifuge tubes, transported to the laboratory on ice packs, and stored temporarily at 4 °C for the isolation and screening of culturable phosphate-solubilizing bacteria. The third subsample was used for metagenomic sequencing; after transport to the laboratory, the soil was freeze-dried at ultra-low temperature and stored at −80 °C until DNA extraction.

### 2.2 Determination of soil physicochemical properties

Soil pH, soil organic matter (SOM), total nitrogen (TN), total phosphorus (TP), total potassium (TK), alkali-hydrolyzable nitrogen (AN), available phosphorus (AP), and available potassium (AK) were determined in safflower rhizosphere soils. Soil pH was measured at a soil-to-water ratio of 1:2.5 by adding 25 mL of distilled water to approximately 10 g of air-dried soil, mixing for 1 min, allowing the suspension to stand for 30 min, and measuring the pH with a PB-10 pH meter (Sartorius, Germany). Soil organic matter was determined by the potassium dichromate volumetric method (Bao, 2000). Total N was measured by the Kjeldahl method, AN by the alkaline hydrolysis diffusion/titration method, TP and AP by the molybdenum-antimony colorimetric method, and TK and AK by flame photometry (Ning and Huang, 2006).

### 2.3 Soil DNA extraction and metagenomic sequencing

Total DNA was extracted from 0.5 g (dry weight) of soil using the E.Z.N.A.^®^ Soil DNA Kit (Omega Bio-tek, USA). DNA concentration and quality were assessed using a NanoDrop-2000 spectrophotometer (Thermo Fisher Scientific, Waltham, MA, USA) and 1% agarose gel electrophoresis, respectively. DNA was randomly fragmented using a Covaris M220 instrument (Gene Corporation, China), and fragments of approximately 400 bp were selected. Paired-end sequencing libraries were constructed using the NEXTFLEX^®^ Rapid DNA-Seq kit (Bioo Scientific, USA). Libraries were sequenced in paired-end mode on an Illumina HiSeq 4000 platform (Illumina Inc., San Diego, CA, USA) by Majorbio Bio-Pharm Technology Co., Ltd. (Shanghai, China).

### 2.4 Sequence quality control, gene prediction, and taxonomic and functional annotation

Raw paired-end reads were quality-filtered using fastp (version 0.23.0). Adapter sequences were removed, and reads with a post-trimming length < 50 bp, an average quality score < 20, or ambiguous bases (N) were discarded. The resulting high-quality reads were assembled using MEGAHIT (version 1.2.9).

Open reading frames (ORFs) were predicted from the assembled contigs using Prodigal (version 2.6.3). Predicted ORFs with nucleotide lengths ≥100 bp were retained and translated into amino-acid sequences. Predicted gene sequences from all samples were clustered using CD-HIT (version 4.6.1) (Fu et al., 2012) at 90% sequence identity and 90% alignment coverage, and the longest sequence in each cluster was retained as the representative sequence to construct the non-redundant gene catalog. High-quality reads from each sample were mapped to the non-redundant gene catalog using SOAPaligner (version 2.21) (Li et al., 2008) at 95% sequence identity, and gene abundances were normalized as reads per kilobase per million mapped reads (RPKM). RPKM-normalized abundances were used for downstream taxonomic and functional comparisons to reduce the influence of differences in gene length and mapped sequencing depth among samples.

For taxonomic annotation, representative protein sequences from the non-redundant gene catalog were aligned against the NCBI non-redundant protein database (NCBI-NR; database snapshot dated 7 October 2024) using DIAMOND (version 2.0.13) in BLASTP mode with an E-value cutoff of 1 × 10^−5^. Taxonomic assignments were determined using the lowest common ancestor (LCA) approach based on the NR taxonomic information. When a sequence could not be unambiguously assigned at a given taxonomic rank, it was retained as unclassified at that rank.

Functional annotation was performed by aligning representative protein sequences against the KEGG database (Kanehisa and Goto, 2000; Kanehisa, 2019; Kanehisa et al., 2025) (database snapshot dated 7 October 2024) using DIAMOND (version 2.0.13) in BLASTP mode with an E-value cutoff of 1 × 10^−5^. When multiple significant KEGG matches were obtained for a query sequence, the best hit was retained as the final functional assignment, and the corresponding KEGG Orthology (KO) term was used for downstream functional analyses.

P-cycling-related genes were identified by exact matching of the resulting KEGG Orthology (KO) annotations against a predefined set of 38 KO terms compiled from published P-cycling/P-transformation gene frameworks. Each KO term was assigned a priori to one of four functional categories according to its reported role in microbial P transformation and its KEGG functional annotation: (i) inorganic P (Pi) dissolution, (ii) organic P (Po) mineralization, (iii) P-starvation response regulation, and (iv) P uptake and transport. Only genes assigned to one of these predefined KO terms were retained as P-cycling-related genes. The 6,019 gene instances therefore represent non-redundant metagenomic gene sequences assigned to these 38 predefined KO terms, rather than 6,019 distinct functional gene types. Here, a “gene instance” refers to one non-redundant metagenomic gene sequence assigned to one of the 38 predefined P-cycling KO terms. Multiple gene instances could be assigned to the same KO and could also share the same taxonomic affiliation. The 460 genus-level bacterial taxa represent the unique genus-level taxonomic assignments obtained from NCBI-NR annotation of these P-cycling-related gene sequences across the complete dataset, rather than 460 independently validated P-cycling genera. Taxonomic affiliations of the selected P-cycling-related genes were subsequently obtained from their NCBI-NR annotations, and the abundances of the associated bacterial taxa and functional genes were used for downstream analyses. The complete KO list, current KEGG descriptions, gene counts, functional-category assignments, and classification basis are provided in Supplementary Table S5. Because shotgun metagenomic sequencing characterizes DNA-level gene presence and abundance, the annotated P-cycling genes were interpreted as indicators of functional potential rather than as evidence of gene expression or actual P-transformation activity in soil.

### 2.5 Isolation, cultivation, and 16S rRNA-based preliminary taxonomic assignment of phosphate-solubilizing bacteria

#### 2.5.1 Isolation and screening of phosphate-solubilizing strains

Ten grams of safflower rhizosphere soil was added to a flask containing glass beads and 90 mL of sterile water and shaken at 28 °C and 150 rpm for 4–6 h to obtain a homogeneous suspension. After standing for 30 min, the resulting 10^−1^ soil suspension was serially diluted tenfold to 10^−6^, and the 10^−4^–10^−6^ dilutions were used for bacterial isolation.

For primary screening, 0.2 mL of each soil dilution was spread on Mongina inorganic phosphate agar medium and incubated at 28 °C for 48 h. Phosphate-solubilizing phenotypes were evaluated based on the clear solubilization zone surrounding each colony. The colony diameter (d) and the total diameter of the clear solubilization zone including the colony (D) were measured through the center of the colony. The phosphate-solubilization index was calculated as SI = D/d, where D represents the total diameter from one outer edge of the clear zone to the opposite outer edge and d represents the colony diameter. A higher SI indicated a more pronounced phosphate-solubilizing phenotype on the medium (Zeng et al., 2023). Colonies forming clear halos were purified by repeated streaking and preserved for taxonomic identification and subsequent selection of a focal isolate for quantitative assessment. Purified strains were maintained on NA slants at 4 °C, and 50% sterile glycerol stocks were prepared and stored at −80 °C for long-term preservation.

*Streptomyces* was selected as the focal genus because it represented the largest proportion of the culturable phosphate-solubilizing isolates and was also identified as a major shared dominant genus in the metagenomic analysis. To select a representative *Streptomyces* strain with a strong plate-based phosphate-solubilizing phenotype, the isolate with the highest SI (1.89) among the *Streptomyces* isolates was selected for quantitative phosphate-solubilization assessment in liquid medium. The liquid assay was therefore intended as a targeted follow-up of a focal *Streptomyces* isolate rather than a quantitative comparison of all 86 isolates. The selected isolate was cultured to the logarithmic growth phase (OD600 = 1.0) and inoculated at 1% (v/v) into 100 mL of sterilized Mongina inorganic phosphate liquid medium. Uninoculated sterile medium served as the blank control. Three independent replicates were established and incubated at 28 °C and 180 rpm for 5 days. A standard curve was generated using potassium dihydrogen phosphate as the standard (y = 0.4946x + 0.125, *r*^2^ = 0.9926). Samples were collected every 24 h, and soluble P concentrations in the culture broth were determined using the molybdenum-antimony colorimetric method. Net phosphate solubilization at each time point was calculated by subtracting the soluble P concentration of the corresponding uninoculated control from that of the LG-4-inoculated culture. The maximum soluble P concentration during the incubation period was recorded (Wang et al., 2020).

#### 2.5.2 Preliminary taxonomic assignment based on the 16S rRNA gene

Purified phosphate-solubilizing strains were streaked on NA agar and incubated at 28 °C for 24 h. Single colonies were selected, and bacterial DNA was extracted using the Chelex-100 method. The 16S rRNA gene was amplified using the extracted DNA as template and the universal primers 27F (5′-AGAGTTTGATCCTGGCTCAG-3′) and 1492R (5′-GGTTACCTTGTTACGACTT-3′), with an expected amplicon size of approximately 1.5 kb (Liu et al., 2022). PCR conditions were as follows: initial denaturation at 94 °C for 4 min; 35 cycles of denaturation at 94 °C for 30 s, annealing at 51 °C for 30 s, and extension at 72 °C for 90 s; followed by a final extension at 72 °C for 10 min and holding at 4 °C. PCR products were purified and sequenced by Tsingke Biotechnology Co., Ltd. (Beijing, China). The resulting sequences were searched against the GenBank database using BLAST and compared with closely related sequences showing high similarity. Multiple sequence alignment was performed using Clustal X, and phylogenetic trees were constructed in MEGA 7.0 using the Neighbor-Joining (NJ) method. Evolutionary distances were calculated using the Maximum Composite Likelihood model, including both transitions and transversions, with uniform rates among sites. Positions containing gaps or missing data were completely deleted, and branch reliability was evaluated using 1,000 bootstrap replicates. Because 16S rRNA gene sequences may not provide sufficient resolution for definitive species-level identification of closely related bacterial taxa, particularly within *Streptomyces*, species-level assignments were treated as provisional. Accordingly, species-level ecological interpretations were not based solely on 16S rRNA gene assignments.

### 2.6 Statistical analysis

Soil physicochemical properties, alpha-diversity indices, and phosphate-solubilization assay data are presented as mean ± standard deviation. One-way analysis of variance (ANOVA) was performed in R 4.5.1 to test overall differences among fields or experimental groups. When the overall ANOVA was significant, Tukey's honestly significant difference (HSD) test was used for *post hoc* pairwise comparisons while controlling the family-wise error rate. Statistical significance was set at *p* < 0.05.

Based on NCBI-NR taxonomic-source annotations of P-cycling functional genes, abundance matrices of P-cycling bacterial taxa were constructed at the phylum and genus levels by summing the RPKM-normalized abundances of genes assigned to each taxon. For presence–absence analysis, a taxon was considered detected in a field when its abundance was >0 in at least one of the three biological replicates. A field-specific taxon was defined as a taxon detected in only one field and absent from all samples of the other six fields. No additional minimum relative-abundance cutoff was applied. Observed richness was calculated as the number of taxa with abundance >0 in each sample. Shannon and Simpson diversity indices were calculated from the relative proportions derived from the RPKM-based taxon abundance matrix. RPKM normalization was used to reduce the effects of differences in gene length and mapped sequencing depth among samples, and no additional rarefaction was applied before alpha-diversity calculation. The three spatially independent biological replicates were nested within each field, and field identity was used as the grouping factor for comparisons among fields and community-level analyses. Principal coordinate analysis (PCoA) based on Bray–Curtis dissimilarities was used to visualize differences in P-cycling bacterial community structure among fields. Differences in community composition among fields were tested using permutational multivariate analysis of variance (PERMANOVA; adonis2, 999 permutations). Homogeneity of multivariate dispersion among fields was assessed using PERMDISP (betadisper) based on the same Bray–Curtis distance matrices, followed by permutation tests (permutest, 999 permutations). PERMANOVA results were reported with degrees of freedom, pseudo-*F* statistics, *R*^2^ values, and permutation-based *p* values.

Spearman rank correlation analysis was used to assess associations between dominant P-cycling bacterial genera or P-cycling functional genes and soil physicochemical properties. Because multiple correlations were tested, raw *p* values were adjusted using the Benjamini–Hochberg false discovery rate (BH-FDR) method, and significance was determined based on adjusted *p* values (*p* < 0.05).

Mantel tests were performed to evaluate associations between the four functional modules (P solubilization, P mineralization, P regulation, and P transport) and soil physicochemical variables, using Bray–Curtis dissimilarity for functional profiles and Euclidean distance for environmental variables, with Spearman correlation and 999 permutations to assess statistical significance.

Random-forest models were constructed in R using the randomForest package, with soil available P (AP) as the response variable and the relative abundances of dominant P-cycling bacterial genera as predictors. The number of trees was set to 1,000, and variable importance was evaluated using the percentage increase in mean squared error (%IncMSE), with higher %IncMSE values indicating a greater predictive contribution of a genus to AP variation. The rfPermute package was further used to conduct 1,000 permutation tests to obtain *p* values for %IncMSE-based variable importance, with *p* < 0.05 considered statistically significant. Model performance was evaluated using out-of-bag (OOB) predictions from the random-forest model. Because the three biological replicates were nested within each of the seven fields, model generalizability was further assessed using leave-one-field-out cross-validation (LOFO-CV) to avoid information leakage between training and validation samples from the same field. In each of seven iterations, all three samples from one field were withheld as the validation set, and the model was trained using the remaining 18 samples from the other six fields. The same predictor set and random-forest settings were used in each iteration. Predictive performance was summarized using the coefficient of determination (*R*^2^), root mean squared error (RMSE), and mean absolute error (MAE). Given the limited sample size (*n* = 21) and the nested sampling structure, with three biological replicates within each of seven fields, the random-forest analysis was used as an exploratory approach to rank the relative predictive importance of bacterial genera rather than as a validated predictive model. More generally, because the Spearman correlation and random-forest analyses were based on 21 sample-level observations nested within seven fields, the observed associations and variable-importance rankings may partly reflect between-field covariation. These analyses were therefore interpreted as exploratory associations and were not considered evidence of field-independent or causal biological relationships.

## 3 Results

### 3.1 Overview of metagenomic sequencing, assembly, and gene prediction

Metagenomic sequencing of the 21 safflower rhizosphere soil samples generated 970,764,770 raw reads totaling 146.59 Gb. Individual samples yielded 41,146,148–53,775,200 raw reads, corresponding to 6.21–8.12 Gb of raw data. After quality control, 954,916,672 clean reads totaling 143.55 Gb were retained. Across the 21 samples, clean read counts ranged from 40,547,612 to 52,846,372 and clean data volumes ranged from 6.10 to 7.94 Gb, indicating relatively consistent sequencing depth among samples. The coefficients of variation for clean read counts and clean data volumes were both approximately 8.2%. Read and base retention rates after quality control ranged from 97.89% to 98.76% and from 97.43% to 98.36%, respectively, with mean values of 98.37% and 97.93%. Sample-specific raw and clean read counts, sequencing data volumes, and retention rates are provided in Supplementary Table S2.

MEGAHIT assembly of the 21 samples generated 1,357,941 contigs with a total assembled length of 919.16 Mb. Individual samples yielded 31,165–101,130 contigs with total assembled lengths of 19.90–70.26 Mb. Mean contig lengths ranged from 621.96 to 758.48 bp; N50 and N90 values ranged from 594 to 668 bp and from 513 to 519 bp, respectively, and maximum contig lengths ranged from 3,087 to 42,204 bp. Gene prediction identified 1,822,686 genes with a total length of 826,269,750 bp and a mean length of 453.33 bp. Screening against the predefined 38-KO framework identified 6,019 P-cycling-related gene instances across the metagenomic dataset, with a total sequence length of 3,186,813 bp and a mean length of 529.46 bp. Sequencing quality-control, assembly, and gene statistics are provided in Supplementary Tables S2–S4. Raw metagenomic sequencing data have been deposited in the NCBI SRA under BioProject accession number PRJNA1517549.

### 3.2 Between-field variation in soil physicochemical properties

Soil physicochemical properties differed significantly among the seven fields (Figures 1A–H, *p* < 0.05). Soil pH was highest in LG (7.8; Figure 1A). SOM and TN were highest in LD, at 28.13 g/kg and 1.76 g/kg, respectively (Figures 1B, C). TP, TK, AN, AP, and AK were highest in XW, at 1.16 g/kg, 29.97 g/kg, 127.67 mg/kg, 64.50 mg/kg, and 480.25 mg/kg, respectively (Figures 1D–H).

**Figure 1:**
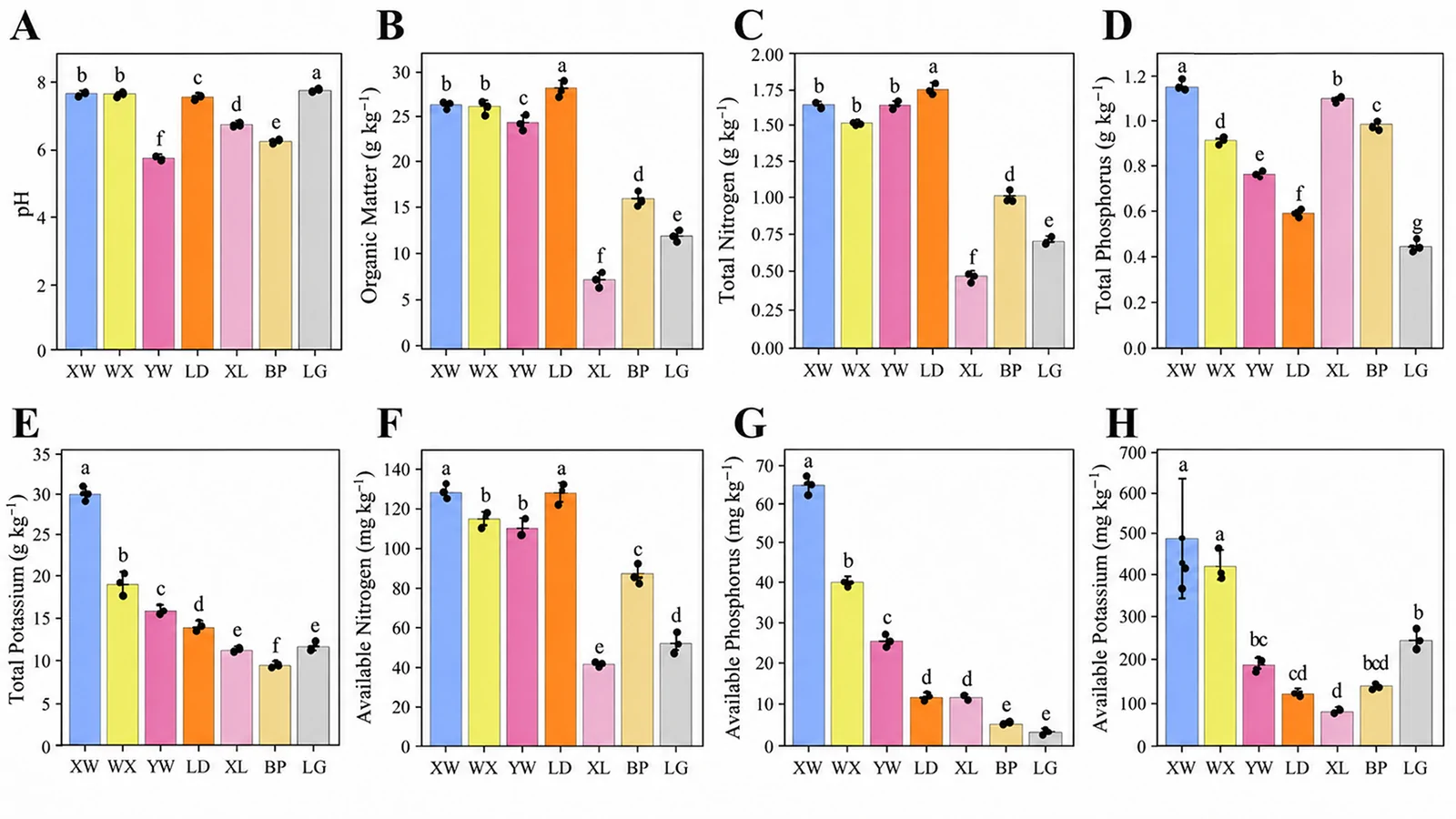
Differences in physicochemical properties of safflower rhizosphere soils among fields. **(A)** pH; **(B)** soil organic matter; **(C)** total nitrogen; **(D)** total phosphorus; **(E)** total potassium; **(F)** alkali-hydrolyzable nitrogen; **(G)** available phosphorus; **(H)** available potassium. Bars represent mean values, error bars indicate SD, and black points represent individual biological replicates (*n* = 3). Different lowercase letters indicate significant differences among fields (*p* < 0.05).

### 3.3 Between-field differentiation in the diversity and overall structure of P-cycling bacterial communities

Alpha-diversity analysis was performed on bacterial taxa obtained from taxonomic-source annotation of P-cycling functional genes. Community richness and diversity differed significantly among fields at both the genus and phylum levels (Table 1, *p* < 0.05). At the genus level, observed richness was higher in BP and XL and lowest in YW, indicating that more genus-level bacterial taxa associated with P-cycling genes were detected in BP and XL. LD had the highest Shannon index and the lowest Simpson index, indicating higher community diversity and lower dominance, whereas BP and XL had lower Shannon indices and higher Simpson indices, indicating relatively lower diversity and greater dominance. At the phylum level, LD had the highest observed richness and Shannon index and the lowest Simpson index, whereas LG had the lowest observed richness and YW had the lowest Shannon index and the highest Simpson index, indicating pronounced between-field differences in phylum-level richness and diversity.

**Table 1:** Alpha-diversity indices of P-cycling bacterial communities among fields.

Sample	Genus level			Phylum level		
	Observed richness (Sobs)	Shannon	Simpson	Observed richness (Sobs)	Shannon	Simpson
BP	233.00 ± 6.08a	3.29 ± 0.02e	0.11 ± 0.00a	31.00 ± 1.00ab	0.84 ± 0.02a	0.59 ± 0.01a
LD	217.67 ± 4.04bc	4.30 ± 0.05a	0.03 ± 0.00e	32.67 ± 0.58a	1.98 ± 0.07b	0.26 ± 0.01b
LG	201.67 ± 5.51de	3.66 ± 0.01b	0.09 ± 0.00b	28.67 ± 0.58b	1.09 ± 0.02c	0.52 ± 0.01c
WX	205.33 ± 7.23cd	3.68 ± 0.01b	0.06 ± 0.00d	29.67 ± 0.58b	0.95 ± 0.01d	0.66 ± 0.01d
XL	229.33 ± 2.31ab	3.26 ± 0.05e	0.11 ± 0.01a	29.67 ± 2.08b	0.82 ± 0.03a	0.61 ± 0.02a
XW	212.67 ± 2.52cd	3.54 ± 0.04c	0.08 ± 0.00c	30.67 ± 0.58ab	1.17 ± 0.02c	0.54 ± 0.01c
YW	189.67 ± 2.52e	3.45 ± 0.01d	0.07 ± 0.00d	29.33 ± 0.58b	0.70 ± 0.02e	0.73 ± 0.01e

Values are presented as mean ± SD (*n* = 3). Different lowercase letters within the same column indicate significant differences among fields (*p* < 0.05, Tukey's HSD test).

PCoA based on Bray–Curtis dissimilarities revealed pronounced field-associated differentiation in P-cycling bacterial community structure. At the phylum level, PC1 and PC2 explained 59.83% and 33.17% of the community variation, respectively, accounting for 93.00% cumulatively (Figure 2B). Samples from XL and XW, and from LG and YW, were positioned relatively close to each other in ordination space, whereas BP, LD, and WX were more clearly separated from the other fields. PERMANOVA confirmed significant differences in phylum-level community structure among fields (pseudo-*F*_6, 14_ = 136.30, *R*^2^ = 0.983, *p* = 0.001). PERMDISP detected no significant difference in multivariate dispersion among fields (*F*_6, 14_ = 2.09, *p*= 0.127), indicating no evidence that the phylum-level PERMANOVA result was driven by heterogeneous within-field dispersion.

**Figure 2:**
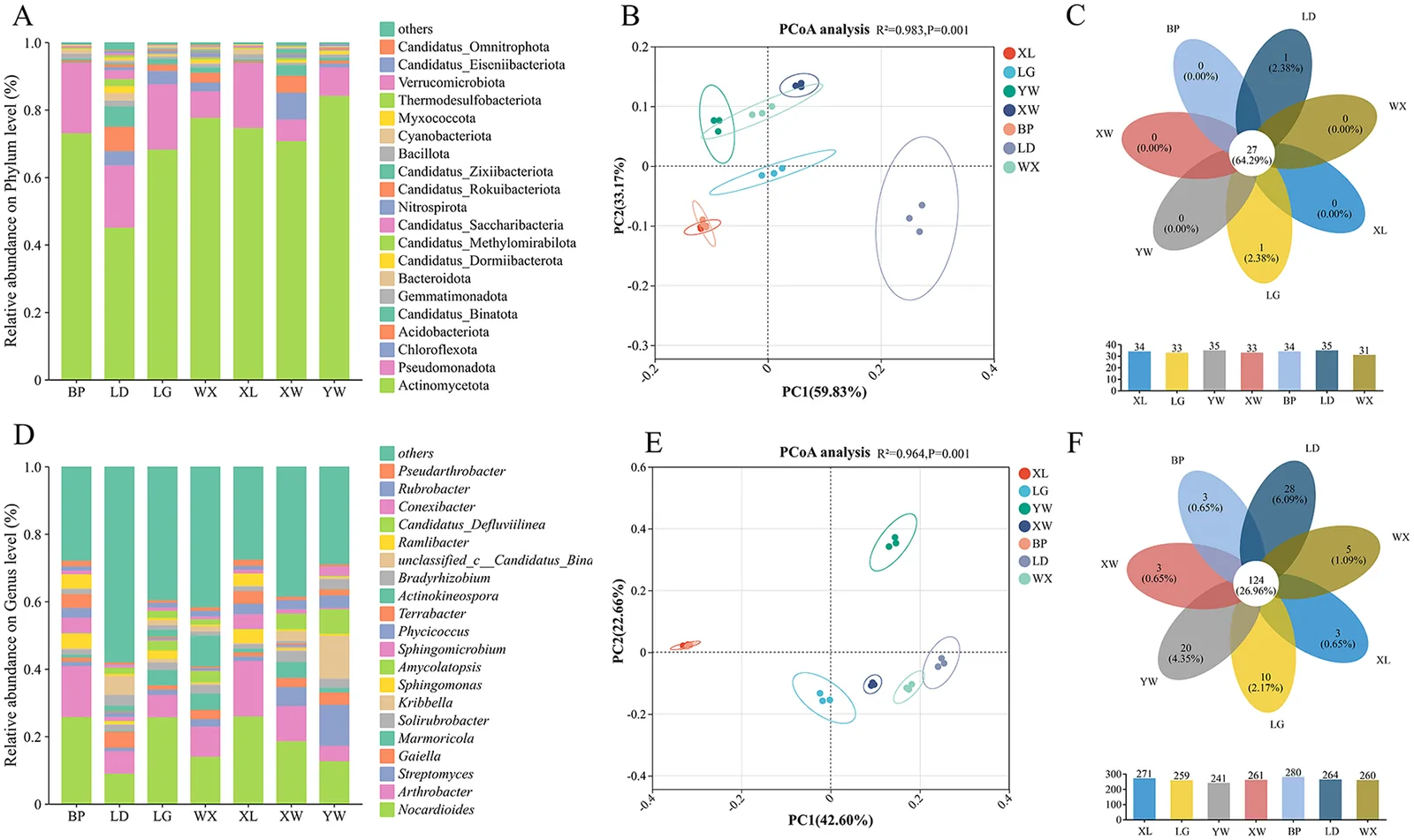
Diversity and composition of P-cycling bacterial communities among fields. **(A)** Relative abundance at the phylum level; **(B)** phylum-level PCoA; **(C)** shared and field-specific phyla; **(D)** relative abundance at the genus level; **(E)** genus-level PCoA; **(F)** shared and field-specific genus-level taxa. For Venn analysis, field-specific taxa were defined as taxa detected in only one field and absent from all samples of the other six fields; no additional minimum relative-abundance cutoff was applied. PCoA was performed based on Bray–Curtis distances, and differences in community structure among fields were tested using PERMANOVA (Adonis; 999 permutations).

At the genus level, PC1 and PC2 explained 42.60% and 22.66% of the community variation, respectively, accounting for 65.26% cumulatively (Figure 2E). Samples from YW, LD, WX, XW, and BP were relatively well separated in ordination space, whereas XL and LG were comparatively close. PERMANOVA likewise indicated significant differences in genus-level community structure among fields (pseudo-*F*_6, 14_ = 62.14, *R*^2^ = 0.964, *p*= 0.001). However, PERMDISP revealed significant heterogeneity in multivariate dispersion among fields (*F*_6, 14_ = 10.39, *p*= 0.002), indicating that the genus-level PERMANOVA result may partly reflect differences in within-field dispersion. Therefore, the pronounced genus-level differentiation among fields was interpreted with caution. Full PERMANOVA and PERMDISP statistics are provided in Supplementary Table S6.

Thus, differences in soil conditions among fields were accompanied by marked changes in the diversity and overall structure of P-cycling bacterial communities. To further identify the taxonomic components underlying these overall differences, dominant, shared, and field-specific P-cycling bacterial taxa were compared at the phylum and genus levels.

### 3.4 Between-field distribution of P-cycling bacterial taxa

A total of 43 P-cycling bacterial phyla were detected. The relatively abundant phyla mainly included Actinomycetota, Pseudomonadota, Chloroflexota, Acidobacteriota, and Candidatus Binatota (Figure 2A). Actinomycetota accounted for relatively high proportions in XL, LG, YW, XW, BP, and WX, with relative abundances ranging from 45% to 85%, but was less abundant in LD. In contrast, Acidobacteriota and Gemmatimonadota showed relatively high abundances in LD, indicating pronounced between-field differences in the distribution of dominant phyla. Venn analysis showed that 27 P-cycling bacterial phyla were shared among all seven fields, with Actinomycetota representing the major shared dominant phylum. LD and LG each contained one field-specific bacterial phylum, whereas no field-specific phyla were detected in the remaining fields (Figure 2C).

At the genus level, a total of 460 bacterial taxa associated with P-cycling genes were detected, including 432 taxa assigned to named genera and 28 taxa that remained unclassified at the genus level. The relatively abundant named genera were mainly *Nocardioides* (0%−25.91%), *Arthrobacter* (0%−16.44%), *Streptomyces* (0%−12.19%), *Marmoricola* (0%−4.84%), and *Gaiella* (0%−4.78%), all belonging to actinobacterial lineages (Figure 2D). *Nocardioides* was relatively abundant in XL, LG, XW, and BP; *Streptomyces* and *Kribbella* were relatively enriched in YW; *Gaiella* and *Bradyrhizobium* were relatively enriched in LD; and *Actinokineospora* showed relatively high abundance in WX. A total of 124 genus-level bacterial taxa were shared among all seven fields. The five most abundant shared named genera were *Nocardioides, Arthrobacter, Streptomyces, Gaiella*, and *Marmoricola*. LD, YW, LG, WX, BP, XL, and XW contained 28, 20, 10, 5, 3, 3, and 3 field-specific genus-level taxa, respectively (Figure 2F).

Overall, P-cycling bacterial communities displayed a spatial pattern characterized by widespread shared dominant taxa together with field-level differentiation of specific taxa. Differences in taxonomic composition do not necessarily correspond directly to differences in P-cycling functions; therefore, the potential for microbial P acquisition, transformation, and utilization was further compared among fields at the functional-gene level.

### 3.5 Between-field variation in P-cycling functional genes

Analysis of P-cycling functional-gene composition showed that major P-cycling genes were detected in all seven fields, although their relative abundances varied among fields (Figure 3). *phoR, phoD, pstA, pstC, pstS, ppx-gppA*, and *phn*-family genes were relatively abundant across the fields and constituted major components of the P-cycling functional-gene profile. Distinct abundance patterns were observed among fields: *phoR* and several *phn*-family genes were relatively abundant in BP and XL; *pst* transport-system genes and *ppx-gppA* were relatively abundant in LG and WX; *phoD* and genes such as *phnJ* and *phnH* accounted for relatively high proportions in YW; multiple gene groups, including *pst, ugp*, and *ppx-gppA*, contributed appreciably in LD, resulting in a relatively dispersed functional composition; and the major P-cycling functional genes showed a comparatively even abundance distribution in XW.

**Figure 3:**
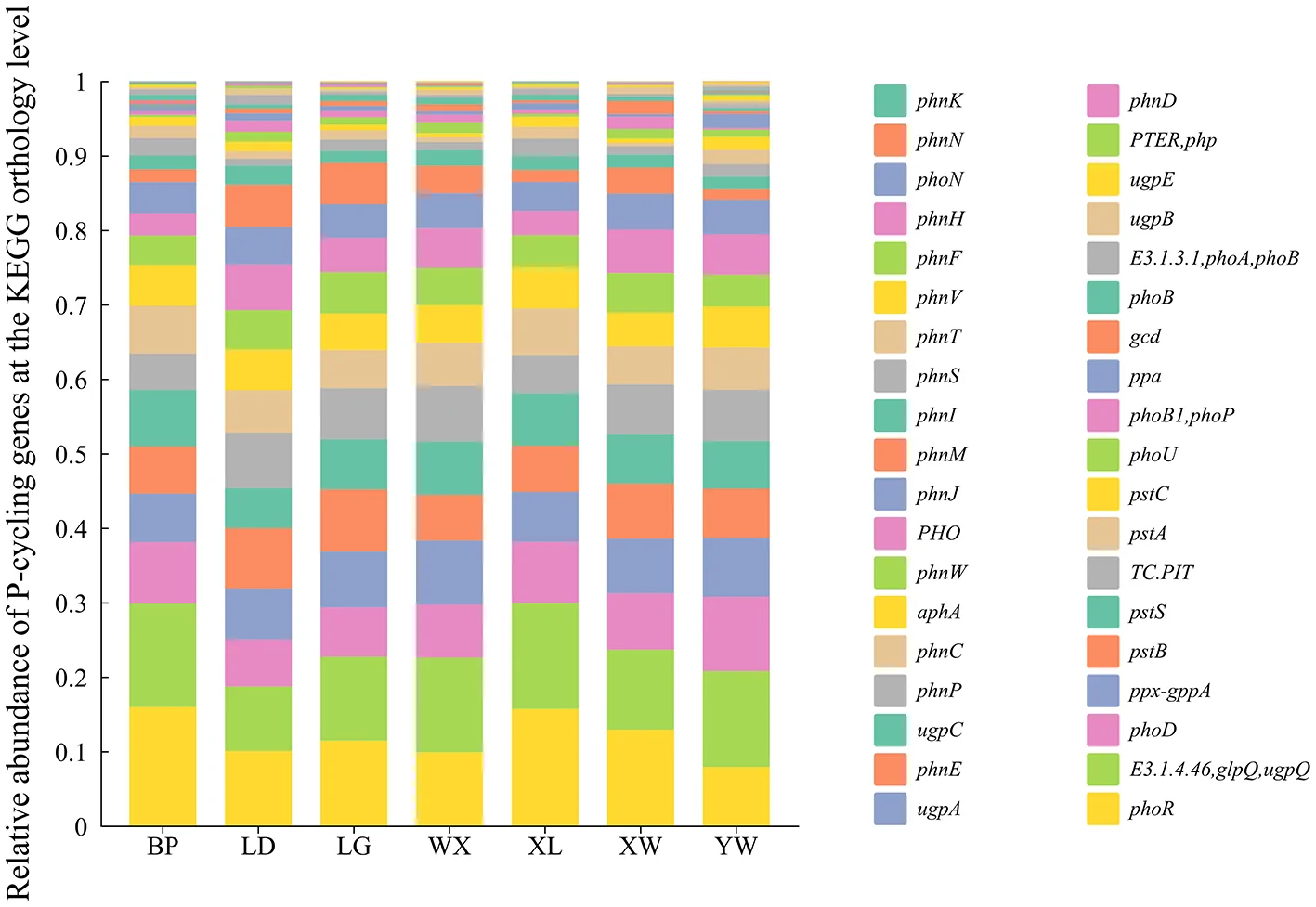
Relative abundance composition of P-cycling functional genes among fields.

These results indicate that all seven fields contained a shared repertoire of genes associated with P solubilization, P mineralization, P regulation, and P transport, whereas the relative abundance profiles of individual functional genes differed among fields, suggesting between-field heterogeneity in the genetic potential for microbial P cycling. These metagenomic patterns represent functional potential and do not demonstrate gene expression or actual P-transformation activity in the soil.

### 3.6 Associations of soil environment with P-cycling bacteria and functional potential

Spearman correlation analysis showed distinct association patterns between dominant P-cycling bacterial genera and soil physicochemical properties (Figure 4A). *Streptomyces* and *Baekduia* were mainly positively correlated with AP, TK, and AK, whereas *Amycolatopsis* and *Marmoricola* showed positive correlations with AP and/or pH. *Gaiella, Agromyces, Candidatus Angelobacter*, and related taxa were positively correlated with TN, SOM, AN, and several readily available nutrient indices. In contrast, *Sphingomonas* and *Ramlibacter* were negatively correlated with TN, SOM, AN, AP, TK, and AK. *Arthrobacter, Pseudarthrobacter*, and *Blastococcus* were positively correlated with TP but negatively correlated with TN and/or SOM, whereas *Nocardioides* was negatively correlated with TN, SOM, AN, TK, and AK.

**Figure 4:**
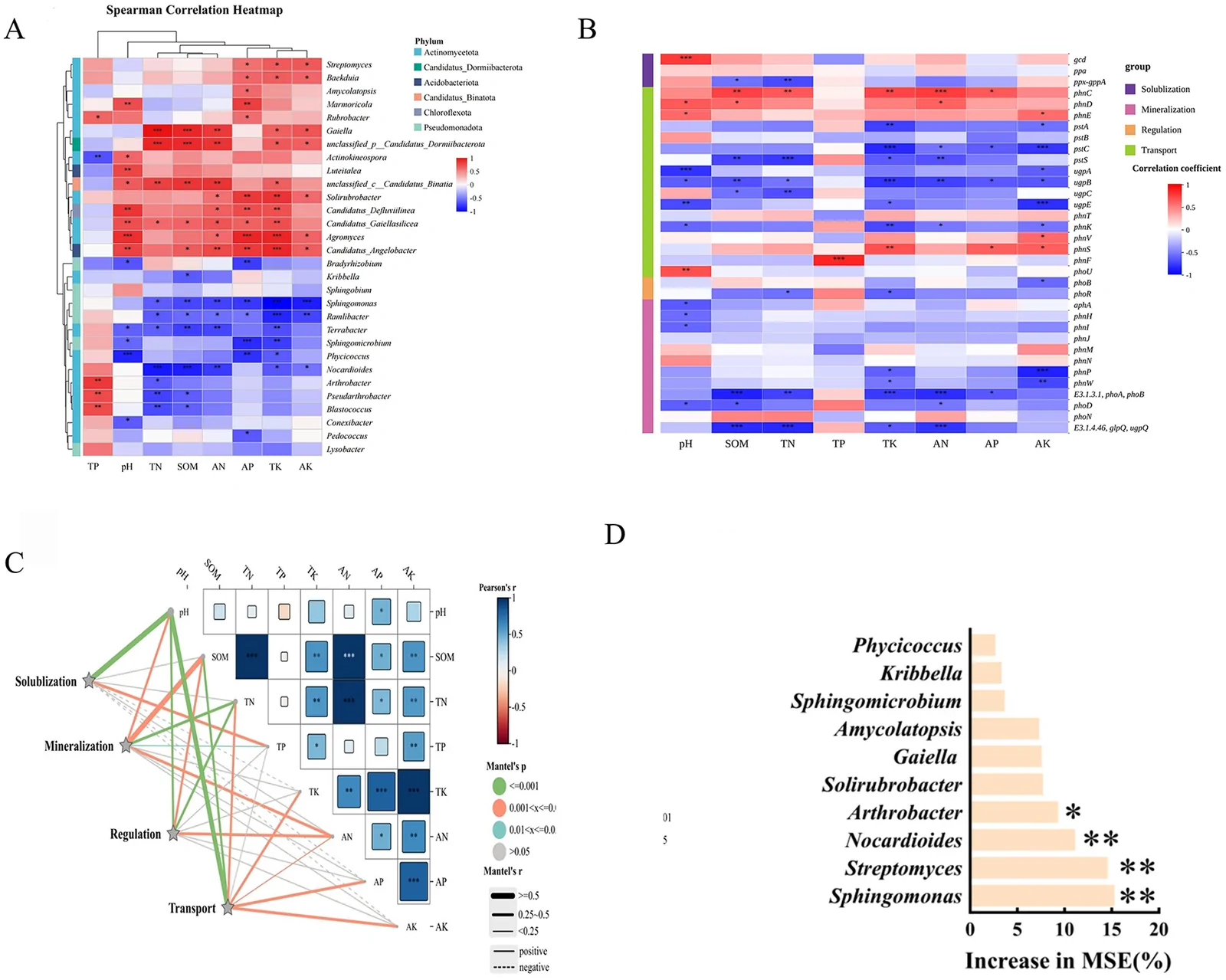
Relationships of soil physicochemical properties with P-cycling bacterial taxa and functional genes. **(A)** Spearman correlations between dominant P-cycling bacterial genera and soil physicochemical properties; **(B)** Spearman correlations between P-cycling functional genes and soil physicochemical properties; **(C)** Mantel tests between the four functional modules (P solubilization, P mineralization, P regulation, and P transport) and soil physicochemical properties; **(D)** variable importance (%IncMSE) of bacterial genera in the exploratory random-forest model using soil available phosphorus (AP) as the response variable. In **A** and **B**, ***p*** values were adjusted for multiple comparisons using the Benjamini–Hochberg false discovery rate (FDR) procedure. ^*^, ^**^, and ^***^ indicate FDR-adjusted *p* values < 0.05, < 0.01, and < 0.001, respectively. In the Mantel test, line color indicates significance level, line width represents the magnitude of the Mantel correlation coefficient, and solid and dashed lines indicate positive and negative correlations, respectively. In **D** ^*^ and ^**^ indicate significant variable importance at *p*< 0.05 and *p*< 0.01, respectively.

At the functional-gene level, Spearman correlation analysis also showed gene-specific association patterns with soil physicochemical properties (Figure 4B). The P-solubilization gene *gcd* was positively correlated with pH, whereas *ppx-gppA* was negatively correlated with SOM and TN. Among P-transport genes, *phnC* was positively correlated with SOM, TN, TK, AN, and AP, whereas *phnS* was positively correlated with TK, AP, and AK. In contrast, several *pst* and *ugp* genes showed negative correlations with soil variables. For example, *pstC* was negatively correlated with TK, AN, AP, and AK, *pstS* with SOM, TN, TK, and AN, and *ugpB* with pH, SOM, TN, TK, AN, AP, and AK. The P-regulation gene *phoR* was negatively correlated with TN and TK. P-mineralization genes also showed contrasting association patterns; for example, *phoD* was negatively correlated with pH, SOM, and AN, whereas *E3.1.3.1, phoA, phoB* was negatively correlated with SOM, TN, TK, AN, and AP. Overall, genes within the same functional module exhibited distinct environmental association patterns.

Mantel tests further revealed differentiated environmental associations at the functional-module level (Figure 4C). pH was associated with all four P-cycling functional modules. The P-solubilization module was associated primarily with pH. In addition to pH, the P-mineralization module was associated with SOM, TN, and AN, whereas the P-regulation and P-transport modules showed associations with pH as well as TK, AN, AP, and AK. Overall, pH showed broad associations with all four P-cycling functional modules, whereas carbon- and nitrogen-related nutrients and readily available nutrients exhibited different patterns of association among functional modules.

Exploratory random-forest analysis ranked *Sphingomonas, Streptomyces, Nocardioides*, and *Arthrobacter* among the genera with relatively high predictive importance for variation in soil AP (Figure 4D). The model showed relatively good apparent performance based on OOB predictions (*R*^2^ = 0.861, RMSE = 7.34 mg/kg, and MAE = 6.25 mg/kg). However, predictive performance decreased substantially under leave-one-field-out cross-validation (*R*^2^ = 0.011, RMSE = 19.61 mg/kg, and MAE = 15.40 mg/kg), indicating limited generalizability to an unseen field. Therefore, these taxa were interpreted as candidate indicators associated with AP variation rather than as robust or independently validated predictors.

### 3.7 Isolation, screening, and identification of culturable phosphate-solubilizing bacteria

Following serial-dilution plating, purification, and preliminary taxonomic assignment based on 16S rRNA gene sequences, 86 bacterial isolates with phosphate-solubilizing phenotypes were obtained. These isolates belonged to three phyla—Firmicutes, Actinobacteria, and Proteobacteria—and represented 45 putative species-level taxa based on 16S rRNA gene sequence analysis (Figures 5A, B). Because 16S rRNA gene sequences may not reliably distinguish closely related bacterial species, these species-level assignments were considered provisional. At the genus level, the isolates mainly included *Priestia, Streptomyces, Stenotrophomonas, Bacillus*, and *Pseudomonas*. *Streptomyces* accounted for the largest proportion (22.1%), followed by *Stenotrophomonas* (11.6%). Representative phosphate-solubilization halos of the cultured isolates are shown in Figure 5C. Among the *Streptomyces* isolates obtained by primary screening, the phosphate-solubilization index (SI) ranged from 1.10 to 1.89. The isolate with the highest SI (1.89), designated *Streptomyces* sp. LG-4 and provisionally affiliated with *S. endophyticus* based on 16S rRNA gene sequence analysis, was selected for quantitative assessment in liquid medium. The soluble P concentration in the LG-4-inoculated culture increased during incubation and reached its maximum on day 3 (Figure 6). After correction for the corresponding uninoculated control, the maximum net phosphate solubilization was 354.60 ± 7.22 mg L^−1^ (*n* = 3), demonstrating the inorganic phosphate-solubilizing capacity of the selected isolate under the tested liquid-culture conditions. Because quantitative assessment was performed only for this selected isolate, this result should not be generalized to other *Streptomyces* isolates or interpreted as evidence of the broader ecological contribution of the genus to soil P cycling.

**Figure 5:**
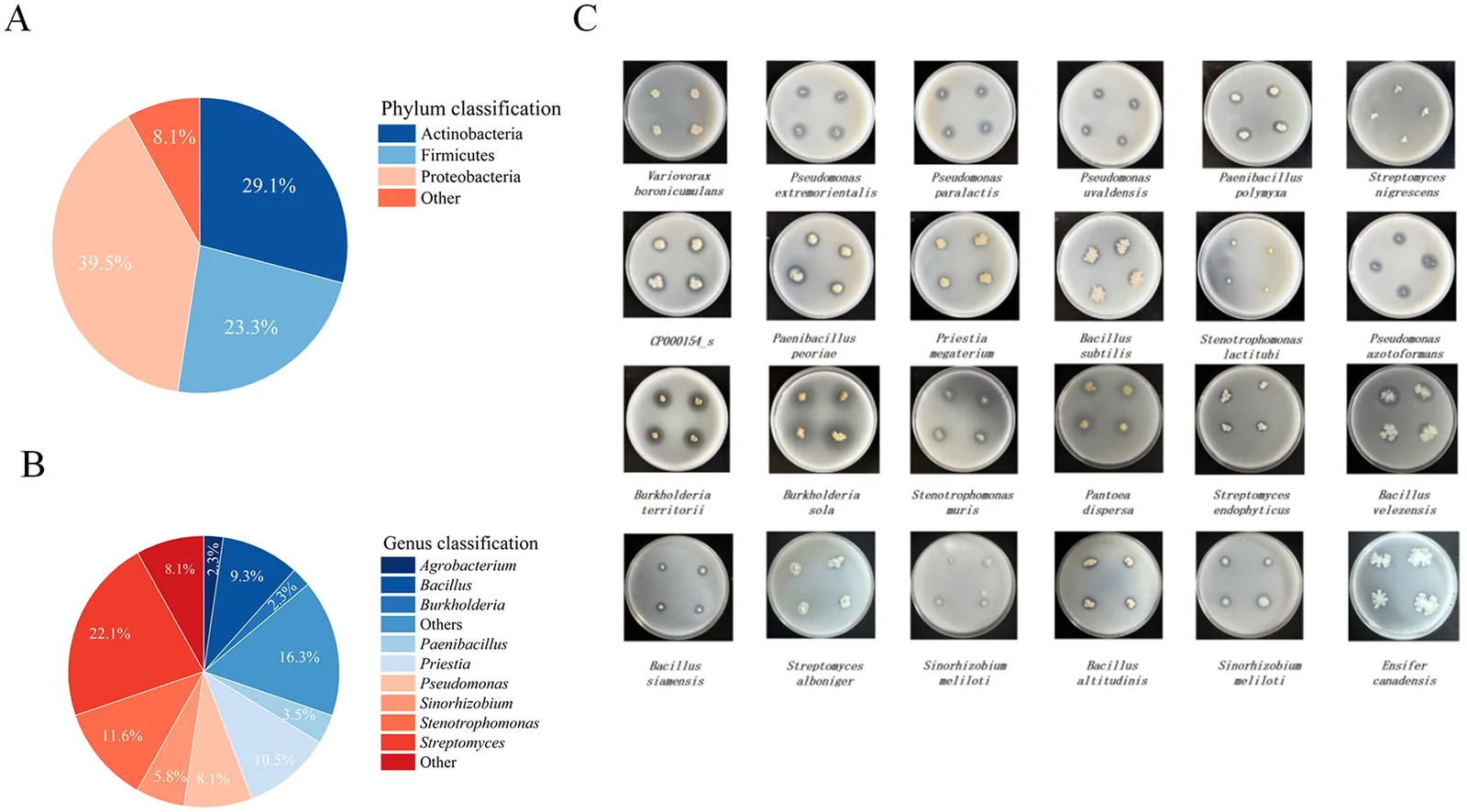
Taxonomic composition and phosphate-solubilizing phenotypes of culturable phosphate-solubilizing bacteria. **(A)** Taxonomic composition at the phylum level; **(B)** taxonomic composition at the genus level; **(C)** phosphate-solubilization halos of representative strains on Mongina inorganic phosphate medium.

**Figure 6:**
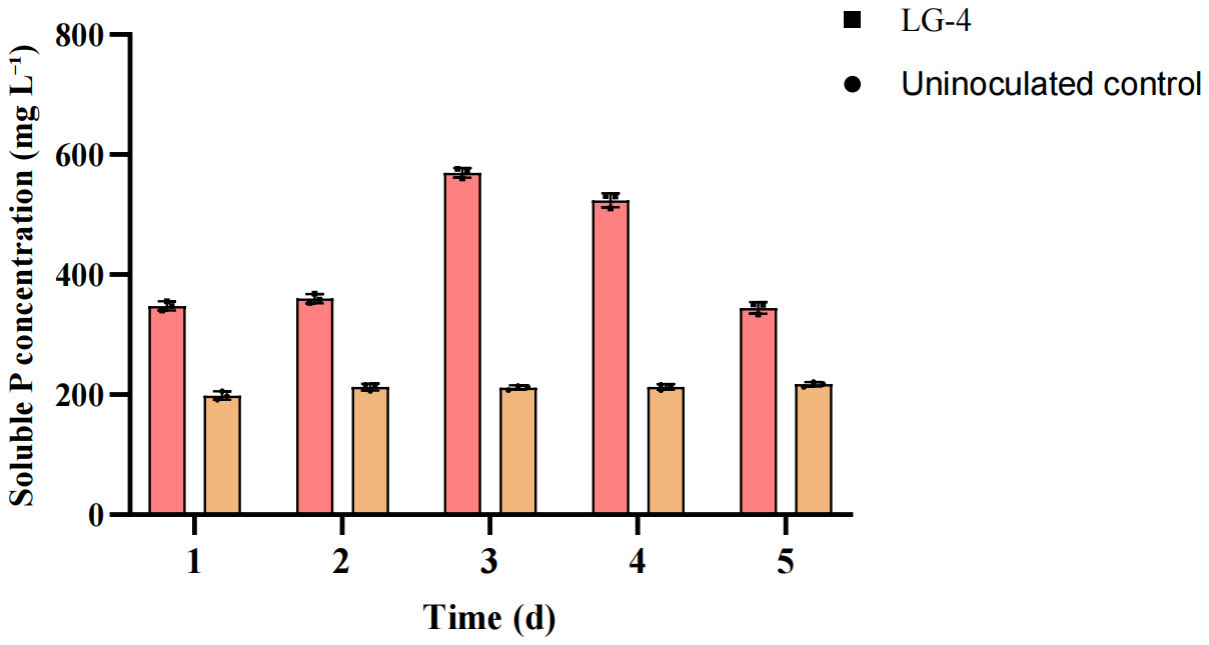
Changes in soluble P concentration in *Streptomyces* sp. LG-4-inoculated and uninoculated control cultures during incubation. *Streptomyces* sp. LG-4 was cultured in Mongina inorganic phosphate liquid medium at 28 °C and 180 rpm for 5 days. Uninoculated sterile medium served as the control. Bars represent mean values, error bars indicate SD, and black points represent individual replicates (*n* = 3).

To further illustrate the phylogenetic relationships among the cultured isolates, representative isolates were selected for construction of a 16S rRNA gene-based Neighbor-Joining phylogenetic tree. The representative isolates were distributed across multiple phylogenetic lineages, reflecting the taxonomic diversity of the culturable phosphate-solubilizing bacteria recovered from these soils (Supplementary Figure S1).

## 4 Discussion

This study showed that soil pH and the status of carbon, nitrogen, phosphorus, and potassium nutrients differed markedly among the seven tobacco–safflower rotation fields and that these differences were accompanied by field-associated differentiation in the diversity, overall structure, and dominant composition of P-cycling bacterial communities. Previous studies have shown that soil chemical properties, particularly pH and P availability, are closely associated with soil bacterial communities and especially with P-cycling microbial assemblages (Lauber et al., 2009; Liu et al., 2018). In the present study, Actinomycetota accounted for relatively high proportions in most fields, and *Nocardioides, Arthrobacter, Streptomyces, Gaiella*, and *Marmoricola* were the major shared dominant genera. At the same time, individual fields contained distinct sets of specific taxa, resulting in a pattern of broadly distributed shared taxa together with field-level differentiation of some genera. Similar spatial differentiation has been reported in other studies of agricultural rhizosphere microbiomes (Lian et al., 2023). The very high PERMANOVA R^2^ values indicated pronounced field-associated structuring of P-cycling bacterial communities. This strong differentiation co-occurred with marked physicochemical heterogeneity among the seven fields, suggesting that field-level environmental variation may contribute to the observed community patterns. At the phylum level, the non-significant PERMDISP result indicated no evidence that the PERMANOVA separation was primarily driven by heterogeneous within-field dispersion. In contrast, significant genus-level dispersion heterogeneity indicated that the genus-level PERMANOVA result may partly reflect differences in within-field variability and should therefore be interpreted cautiously. Accordingly, the observed patterns are best interpreted as strong between-field community differentiation occurring in the context of substantial environmental heterogeneity. Given the cross-sectional design and the absence of non-rotation controls, the relative contributions of individual environmental factors and the rotation system cannot be disentangled, and these patterns should not be interpreted as causal effects of the tobacco–safflower rotation system.

Despite differences in the taxonomic composition of P-cycling bacteria among fields, genes related to P solubilization, P mineralization, P regulation, and P transport were detected in all seven fields, indicating a shared basic P-cycling functional composition in the rhizosphere microbiome of the study area. At the same time, the relative abundances of genes such as *phoR, phoD, pst, phn, ugp*, and *ppx-gppA* differed among fields, suggesting that P-cycling functional potential was not identical across sites and was reflected mainly by variation in the relative abundance profiles of genes involved in P acquisition, transformation, and regulation. Previous studies have reported that microbial taxonomic composition can differ substantially among environments while overall functional composition remains comparatively stable (Lian et al., 2023; Chen et al., 2022). Metagenomic comparisons across different land-use types have likewise shown that functions related to P mineralization, inorganic P solubilization, and P-limitation responses are widespread across soils, although the potential contributions of the associated functional genes vary (Siles et al., 2022). Soil P supply and long-term nutrient management have also been shown to alter the abundances of P-cycling genes including *phoD, pst, phn*, and *phoR* (Bergkemper et al., 2016; Dai et al., 2020; Liu L. et al., 2023). Such differences in functional-gene abundance may reflect variation in microbial P acquisition and transformation potential under contrasting soil nutrient conditions. However, because gene abundances were normalized for gene length and mapped sequencing depth using RPKM, but not normalized to taxon-specific genome equivalents or universal single-copy marker genes, variation in functional-gene abundance may also partly reflect changes in the abundance of microorganisms carrying these genes. Therefore, the present analysis cannot fully distinguish community-compositional shifts from changes in functional potential per microbial genome.

Environmental association analyses further showed that different P-cycling bacterial genera and functional genes displayed distinct relationships with soil physicochemical properties, with several genus–environment and gene–environment associations remaining significant after correction for multiple comparisons. At the functional-module level, pH showed broad associations with all four P-cycling functional modules. Soil pH is closely associated with microbial community structure and can also affect the adsorption, precipitation, and dissolution of P with minerals containing Ca, Fe, and Al, thereby altering soil P availability (Lauber et al., 2009; Hinsinger, 2001). Previous studies similarly identified pH and available P as important environmental factors associated with the distribution of *phoD*-harboring microorganisms and other P-cycling functional genes (Liu et al., 2018; Ragot et al., 2015). In addition, SOM, TN, AN, and readily available nutrients showed differentiated associations with the various P-cycling modules, suggesting complex associations between soil P-cycling functional potential and carbon, nitrogen, and other nutrient conditions. Metagenomic studies have also identified soil AP and TN as important environmental factors closely associated with P-transport and organic-P-mineralization genes and their related microbial taxa (Liao et al., 2023). Given the strong field-associated differentiation revealed by the PERMANOVA analyses, some of the observed associations among soil physicochemical properties, microbial taxa, functional-gene profiles, and AP may reflect shared between-field structure rather than independent sample-level biological relationships. Because these variables varied simultaneously among fields, the associations observed in the present study should be interpreted as statistical covariation rather than evidence of causal relationships. Accordingly, pH and the candidate bacterial genera identified here are more appropriately considered variables associated with between-field variation rather than demonstrated drivers of P-cycling processes or soil AP.

Exploratory random-forest analysis ranked *Sphingomonas, Streptomyces, Nocardioides*, and *Arthrobacter* among the genera with relatively high predictive importance for variation in soil AP. Although OOB predictions indicated relatively good apparent model performance, predictive performance decreased markedly under leave-one-field-out cross-validation, indicating limited generalizability across fields. This contrast suggests that the model may capture substantial field-associated structure within the present dataset but does not provide robust evidence of field-independent predictive relationships. Accordingly, these genera are more appropriately considered candidate indicator taxa associated with AP variation rather than validated predictors, and their ecological relevance requires validation using larger independent datasets and targeted experiments. A long-term P-fertilization study likewise identified *Nocardioides* and *Gaiella* as important P-cycling taxa, with *Nocardioides* representing one of the major taxa associated with the inorganic P-solubilization gene *gcd* (Liu et al., 2024). In addition, *Streptomyces* was a major shared dominant genus across the sampled fields, and previous studies have reported that many *Streptomyces* strains can solubilize inorganic P and mineralize organic P through the production of organic acids and the secretion of phosphatases or phytases (Chouyia et al., 2022; Jog et al., 2014). These observations support further investigation of *Streptomyces* as a candidate P-cycling group, but do not establish a causal role in determining soil AP.

Culture-dependent analysis yielded 86 bacterial isolates with phosphate-solubilizing phenotypes, with *Streptomyces* accounting for the largest proportion (22.1%). Independently, *Streptomyces* was also a major shared dominant genus in the metagenomic analysis and ranked among the genera with relatively high predictive importance for AP in the exploratory random-forest analysis. These concordant observations identify *Streptomyces* as a candidate group worthy of further investigation, but they should be regarded as complementary rather than as independent validation of the same P-cycling mechanism. Previous studies integrating culturable phosphate-solubilizing bacteria with analyses of P-cycling functional genes such as *gcd* and *bpp* have similarly shown that culture-derived microbial resources and functional-gene information can provide complementary perspectives on soil P-acquisition potential (He and Wan, 2022). In addition, many *Streptomyces* strains have been reported to possess phosphate-solubilizing and plant growth-promoting potential (Chouyia et al., 2022; Jog et al., 2014; Vargas Hoyos et al., 2021). However, plant-growth promotion was not evaluated in the present study, and the performance of the cultured isolates under soil, pot, or field conditions remains unknown. In the present study, however, the cultured *Streptomyces* isolates cannot be directly linked to specific P-cycling genes or strain-level taxonomic sources identified in the metagenomic dataset. Moreover, quantitative phosphate-solubilization assessment was performed only for the selected focal *Streptomyces* isolate with the highest SI. Its maximum net phosphate solubilization of 354.60 ± 7.22 mg L^−1^ demonstrates phosphate-solubilizing capacity under the tested laboratory conditions, but does not establish that other *Streptomyces* isolates have comparable activity or that the genus makes a major ecological contribution to P transformation *in situ*. Therefore, the culture-based result should be regarded as strain-level functional evidence rather than genus-level ecological validation, and further studies linking cultured strains with specific functional genes and evaluating their activity under soil or field conditions are required.

This study has several limitations. First, no tobacco continuous-cropping, safflower monoculture, or alternative rotation controls were included. Therefore, the results primarily reflect between-field differences and environmental associations among tobacco–safflower rotation fields and cannot be directly attributed to the rotation system itself. Second, the random-forest analysis was based on only 21 samples; therefore, the stability and generalizability of genus-level importance rankings remain uncertain and should be validated using larger independent datasets. Third, metagenomic annotation reflects the presence and abundance of functional genes, i.e., functional potential, but does not directly represent gene-expression levels or actual rates of P transformation. Fourth, species-level assignments of the cultured isolates were based on 16S rRNA gene sequences and should therefore be considered provisional, particularly for closely related taxa within *Streptomyces*; genome-based taxonomic analyses will be required for definitive species-level identification. Fifth, selective culture media recover only a subset of culturable phosphate-solubilizing bacteria, and no direct correspondence can currently be established between the cultured isolates and specific P-cycling genes or strain-level taxonomic sources identified in the metagenomic dataset. Sixth, quantitative phosphate-solubilization assessment was performed for only one selected *Streptomyces* isolate with the highest SI; therefore, its *in vitro* performance cannot be generalized to other *Streptomyces* isolates or to the genus as a whole, nor can it be considered direct evidence of the broader ecological importance of *Streptomyces* in soil P cycling. The present study did not evaluate plant-growth promotion or the performance of the isolated strains under soil, pot, or field conditions; therefore, no direct conclusions regarding their agricultural effectiveness can yet be drawn. Future studies could integrate soil P fractionation, phosphatase activity, quantitative analysis of functional genes, and metatranscriptomics, together with whole-genome sequencing and genome-based taxonomic analyses of representative cultured isolates. Functional validation using multiple representative isolates, soil microcosms, pot experiments, and field trials will also be required to determine whether the observed *in vitro* phosphate-solubilizing phenotypes translate into effects on plant P acquisition, plant growth, or performance under soil and field conditions.

## Conclusion

This study showed that soil pH was associated with between-field differences in P-cycling bacterial communities and metagenomically inferred functional potential across tobacco–safflower rotation fields. Exploratory random-forest analysis identified *Sphingomonas, Streptomyces, Nocardioides*, and *Arthrobacter* as candidate indicator taxa associated with variation in soil available P. Culture-dependent isolation further showed that *Streptomyces* sp. LG-4 had phosphate-solubilizing capacity under laboratory conditions and warrants further taxonomic, functional, and ecological validation.

## Data Availability

The datasets presented in this study can be found in online repositories. The names of the repository/repositories and accession number(s) can be found in the article/Supplementary material.
