# Supplementary material for: Metagenomic and culture-dependent characterization of between-field variation in phosphorus-cycling microbial communities and functional potential across tobacco–safflower rotation fields
Source: Front Microbiol. 2026 Oct 8;17:1983930. doi: 10.3389/fmicb.2026.1983930 (PMC13686992; doi:10.3389/fmicb.2026.1983930)
Supplement: Supplementary file 1 [file Table_1.docx]

Supplementary Table S1. Information on sampling sites for the seven flue-cured tobacco–safflower rotation fields

| Sample number | Group | Sampling date | Sampling area | Altitude (meters) | Temperature(℃） | Relative humidity |
| --- | --- | --- | --- | --- | --- | --- |
| SC-YW | SC | 2025-4-25 | China:Lijiang(26.77243864 N100.66657950 E) | 1638.14 | 21.4 | 80% |
| SC-XW | SC | 2025-4-25 | China:Lijiang(26.70331264 N100.70721921 E) | 1604.04 | 21.4 | 80% |
| SC-WX | SC | 2025-4-25 | China:Lijiang(26.68272866 N100.70193159 E) | 1753.43 | 21.4 | 80% |
| QN-BP | QN | 2025-4-25 | China:Lijiang(26.31116937 N100.57716172 E) | 1605.01 | 21.4 | 80% |
| QN-XL | QN | 2025-4-25 | China:Lijiang(26.30033840 N100.57991601 E) | 1519.17 | 21.4 | 80% |
| QN-LG | QN | 2025-4-25 | China:Lijiang(26.28466031 N100.61314341 E) | 1380.00 | 21.4 | 80% |
| LD-LD | LD | 2025-4-25 | China:Lijiang(26.55729537 N100.85006854 E) | 1770.31 | 21.4 | 80% |

Supplementary Table S2. Summary of raw and clean metagenomic sequencing data for the 21 safflower rhizosphere soil samples

| **Sample** | **Insert size (bp)** | **Raw reads** | **Raw bases (Gb)** | **Clean reads** | **Clean bases (Gb)** | **Reads retained (%)** | **Bases retained (%)** |
| --- | --- | --- | --- | --- | --- | --- | --- |
| BP_1 | 443 | 49,727,952 | 7.509 | 49,088,474 | 7.379 | 98.714 | 98.274 |
| BP_2 | 456 | 46,402,296 | 7.007 | 45,637,900 | 6.860 | 98.353 | 97.909 |
| BP_3 | 452 | 50,696,052 | 7.655 | 49,851,344 | 7.492 | 98.334 | 97.875 |
| LD_1 | 449 | 41,994,690 | 6.341 | 41,423,412 | 6.230 | 98.640 | 98.239 |
| LD_2 | 457 | 43,465,422 | 6.563 | 42,925,802 | 6.456 | 98.759 | 98.361 |
| LD_3 | 454 | 47,502,504 | 7.173 | 46,856,522 | 7.046 | 98.640 | 98.230 |
| LG_1 | 450 | 41,520,808 | 6.270 | 40,774,784 | 6.130 | 98.203 | 97.765 |
| LG_2 | 451 | 44,747,028 | 6.757 | 43,988,402 | 6.613 | 98.305 | 97.872 |
| LG_3 | 450 | 44,714,206 | 6.752 | 43,963,124 | 6.610 | 98.320 | 97.892 |
| WX_1 | 458 | 41,146,148 | 6.213 | 40,547,612 | 6.098 | 98.545 | 98.142 |
| WX_2 | 452 | 44,476,490 | 6.716 | 43,627,902 | 6.557 | 98.092 | 97.627 |
| WX_3 | 453 | 46,748,872 | 7.059 | 46,038,098 | 6.920 | 98.480 | 98.034 |
| XL_1 | 450 | 49,307,172 | 7.445 | 48,511,956 | 7.291 | 98.387 | 97.930 |
| XL_2 | 439 | 41,464,460 | 6.261 | 40,718,588 | 6.120 | 98.201 | 97.746 |
| XL_3 | 458 | 53,775,200 | 8.120 | 52,846,372 | 7.942 | 98.273 | 97.808 |
| XW_1 | 457 | 47,436,820 | 7.163 | 46,593,156 | 7.004 | 98.222 | 97.775 |
| XW_2 | 452 | 51,696,892 | 7.806 | 50,808,872 | 7.636 | 98.282 | 97.826 |
| XW_3 | 450 | 47,233,166 | 7.132 | 46,451,504 | 6.982 | 98.345 | 97.900 |
| YW_1 | 454 | 42,401,012 | 6.403 | 41,505,992 | 6.238 | 97.889 | 97.433 |
| YW_2 | 453 | 42,983,324 | 6.490 | 42,279,604 | 6.356 | 98.363 | 97.927 |
| YW_3 | 456 | 51,324,256 | 7.750 | 50,477,252 | 7.587 | 98.350 | 97.904 |

Note: Raw reads and clean reads both refer to the number of paired-end sequencing reads; data volume was calculated using 1 Gb = 10⁹ bp.

Supplementary Table S3. Summary of metagenomic assembly statistics for the 21 safflower rhizosphere soil samples

| **Sample** | **Contig number** | **Total length (bp)** | **Total length (Mb)** | **Mean length (bp)** | **N50 (bp)** | **N90 (bp)** | **Maximum length (bp)** | **Minimum length (bp)** |
| --- | --- | --- | --- | --- | --- | --- | --- | --- |
| BP_1 | 84,730 | 54,874,895 | 54.875 | 647.64 | 616 | 516 | 7,025 | 500 |
| BP_2 | 75,118 | 48,378,982 | 48.379 | 644.04 | 614 | 516 | 10,541 | 500 |
| BP_3 | 83,386 | 53,998,051 | 53.998 | 647.57 | 616 | 516 | 7,730 | 500 |
| LD_1 | 37,208 | 23,141,780 | 23.142 | 621.96 | 594 | 513 | 7,405 | 500 |
| LD_2 | 41,601 | 25,964,328 | 25.964 | 624.13 | 595 | 513 | 3,087 | 500 |
| LD_3 | 50,932 | 31,840,193 | 31.840 | 625.15 | 596 | 513 | 41,059 | 500 |
| LG_1 | 31,165 | 19,902,295 | 19.902 | 638.61 | 605 | 514 | 42,177 | 500 |
| LG_2 | 36,472 | 23,344,415 | 23.344 | 640.06 | 607 | 515 | 42,070 | 500 |
| LG_3 | 33,297 | 21,035,743 | 21.036 | 631.76 | 600 | 514 | 42,204 | 500 |
| WX_1 | 52,047 | 39,476,578 | 39.477 | 758.48 | 668 | 519 | 26,589 | 500 |
| WX_2 | 58,318 | 43,864,249 | 43.864 | 752.16 | 662 | 519 | 31,824 | 500 |
| WX_3 | 64,033 | 48,291,023 | 48.291 | 754.16 | 663 | 519 | 39,948 | 500 |
| XL_1 | 84,087 | 54,522,187 | 54.522 | 648.40 | 616 | 516 | 13,029 | 500 |
| XL_2 | 62,910 | 40,316,490 | 40.316 | 640.86 | 611 | 515 | 6,112 | 500 |
| XL_3 | 95,277 | 61,923,771 | 61.924 | 649.93 | 618 | 516 | 10,378 | 500 |
| XW_1 | 70,154 | 49,827,957 | 49.828 | 710.27 | 645 | 518 | 17,303 | 500 |
| XW_2 | 77,777 | 55,022,832 | 55.023 | 707.44 | 644 | 518 | 23,129 | 500 |
| XW_3 | 66,291 | 47,436,927 | 47.437 | 715.59 | 646 | 518 | 24,975 | 500 |
| YW_1 | 77,132 | 53,810,163 | 53.810 | 697.64 | 647 | 519 | 20,333 | 500 |
| YW_2 | 74,876 | 51,926,967 | 51.927 | 693.51 | 643 | 518 | 12,609 | 500 |
| YW_3 | 101,130 | 70,259,185 | 70.259 | 694.74 | 645 | 519 | 23,755 | 500 |

Note: Only contigs with lengths ≥500 bp were included. Mean length was calculated by dividing the total contig length by the number of contigs.

Supplementary Table S4. Statistics of predicted genes and phosphorus-cycling-related genes

| **Gene category** | **Gene number** | **Total length (bp)** | **Average length (bp)** |
| --- | --- | --- | --- |
| All predicted genes | 1,822,686 | 826,269,750 | 453.33 |
| Phosphorus-cycling-related genes | 6,019 | 3,186,813 | 529.46 |

Note: The 6,019 genes represent P-cycling-related genes obtained after functional-gene screening and annotation, rather than the complete non-redundant gene catalog.

Supplementary Table S5. KEGG Orthology (KO) terms used for identification and functional classification of phosphorus-cycling genes

The candidate P-cycling gene set was predefined from published P-cycling/P-transformation gene frameworks and grouped into four functional categories. KO identifiers and current functional descriptions were cross-checked against the KEGG database snapshot dated 7 October 2024.

| Functional category | Gene | KO number | KEGG description (20241007) | Gene count | Classification basis |
| --- | --- | --- | --- | --- | --- |
| Inorganic P (Pi) dissolution | *gcd* | K00117 | quinoprotein glucose dehydrogenase [EC:1.1.5.2] | 254 | Jiang et al. (2023); KEGG 20241007 checked |
| Inorganic P (Pi) dissolution | *ppa* | K01507 | inorganic pyrophosphatase [EC:3.6.1.1] | 159 | Jiang et al. (2023); KEGG 20241007 checked |
| Inorganic P (Pi) dissolution | *ppx-gppA* | K01524 | exopolyphosphatase / guanosine-5'-triphosphate,3'-diphosphate pyrophosphatase [EC:3.6.1.11 3.6.1.40] | 495 | Jiang et al. (2023); KEGG 20241007 checked |
| Organic P (Po) mineralization | *aphA* | K00897 | kanamycin kinase [EC:2.7.1.95] | 16 | Jiang et al. (2023) framework; current KEGG 20241007 annotation differs (see note) |
| Organic P (Po) mineralization | PHO | K01078 | acid phosphatase [EC:3.1.3.2] | 5 | Published P-transformation framework; KEGG 20241007 checked |
| Organic P (Po) mineralization | *phoD* | K01113 | alkaline phosphatase D [EC:3.1.3.1] | 477 | Jiang et al. (2023); KEGG 20241007 checked |
| Organic P (Po) mineralization | *phoN* | K09474 | acid phosphatase (class A) [EC:3.1.3.2] | 1 | Jiang et al. (2023); KEGG 20241007 checked |
| Organic P (Po) mineralization | *phoA/phoB* | K01077 | alkaline phosphatase [EC:3.1.3.1] | 116 | Jiang et al. (2023); KEGG 20241007 checked |
| Organic P (Po) mineralization | *glpQ/ugpQ* | K01126 | glycerophosphoryl diester phosphodiesterase [EC:3.1.4.46] | 768 | Jiang et al. (2023); KEGG 20241007 checked |
| Organic P (Po) mineralization | *phnW* | K03430 | 2-aminoethylphosphonate-pyruvate transaminase [EC:2.6.1.37] | 12 | Jiang et al. (2023); KEGG 20241007 checked |
| Organic P (Po) mineralization | *phnP* | K06167 | phosphoribosyl 1,2-cyclic phosphate phosphodiesterase [EC:3.1.4.55] | 51 | Jiang et al. (2023); KEGG 20241007 checked |
| Organic P (Po) mineralization | *phnH* | K06165 | alpha-D-ribose 1-methylphosphonate 5-triphosphate synthase subunit PhnH [EC:2.7.8.37] | 2 | Jiang et al. (2023); KEGG 20241007 checked |
| Organic P (Po) mineralization | *phnI* | K06164 | alpha-D-ribose 1-methylphosphonate 5-triphosphate synthase subunit PhnI [EC:2.7.8.37] | 4 | Jiang et al. (2023); KEGG 20241007 checked |
| Organic P (Po) mineralization | *phnJ* | K06163 | alpha-D-ribose 1-methylphosphonate 5-phosphate C-P lyase [EC:4.7.1.1] | 3 | Jiang et al. (2023); KEGG 20241007 checked |
| Organic P (Po) mineralization | *phnM* | K06162 | alpha-D-ribose 1-methylphosphonate 5-triphosphate diphosphatase [EC:3.6.1.63] | 8 | Jiang et al. (2023); KEGG 20241007 checked |
| Organic P (Po) mineralization | *phnN* | K05774 | ribose 1,5-bisphosphokinase [EC:2.7.4.23] | 1 | Jiang et al. (2023); KEGG 20241007 checked |
| Organic P (Po) mineralization | *PTER/php* | K07048 | phosphotriesterase-related protein | 62 | Published P-transformation framework; KEGG 20241007 checked |
| P-starvation response regulation | *phnF* | K02043 | GntR family transcriptional regulator, phosphonate transport system regulatory protein | 2 | Published P-transformation framework; KEGG 20241007 checked |
| P-starvation response regulation | *phoR* | K07636 | two-component system, OmpR family, phosphate regulon sensor histidine kinase PhoR [EC:2.7.13.3] | 845 | Jiang et al. (2023); KEGG 20241007 checked |
| P-starvation response regulation | *phoU* | K02039 | phosphate transport system protein | 257 | KEGG 20241007 phosphate/phosphonate-system annotation |
| P-starvation response regulation | *phoB1/phoP* | K07658 | two-component system, OmpR family, alkaline phosphatase synthesis response regulator PhoP | 288 | KEGG 20241007 phosphate/phosphonate-system annotation |
| P-starvation response regulation | *phoB* | K07657 | two-component system, OmpR family, phosphate regulon response regulator PhoB | 122 | Jiang et al. (2023); KEGG 20241007 checked |
| P uptake and transport | *pstA* | K02038 | phosphate transport system permease protein | 318 | Jiang et al. (2023); KEGG 20241007 checked |
| P uptake and transport | *pstB* | K02036 | phosphate transport system ATP-binding protein [EC:7.3.2.1] | 294 | Jiang et al. (2023); KEGG 20241007 checked |
| P uptake and transport | *phnV* | K11082 | 2-aminoethylphosphonate transport system permease protein | 5 | KEGG 20241007 phosphate/phosphonate-system annotation |
| P uptake and transport | *phnK* | K05781 | putative phosphonate transport system ATP-binding protein | 1 | KEGG 20241007 phosphate/phosphonate-system annotation |
| P uptake and transport | *ugpB* | K05813 | sn-glycerol 3-phosphate transport system substrate-binding protein | 76 | Jiang et al. (2023); KEGG 20241007 checked |
| P uptake and transport | *ugpC* | K05816 | sn-glycerol 3-phosphate transport system ATP-binding protein [EC:7.6.2.10] | 43 | Jiang et al. (2023); KEGG 20241007 checked |
| P uptake and transport | *phnS* | K11081 | 2-aminoethylphosphonate transport system substrate-binding protein | 8 | Published P-transformation framework; KEGG 20241007 checked |
| P uptake and transport | *phnD* | K02044 | phosphonate transport system substrate-binding protein | 50 | Jiang et al. (2023); KEGG 20241007 checked |
| P uptake and transport | *phnE* | K02042 | phosphonate transport system permease protein | 34 | Jiang et al. (2023); KEGG 20241007 checked |
| P uptake and transport | *ugpA* | K05814 | sn-glycerol 3-phosphate transport system permease protein | 46 | Jiang et al. (2023); KEGG 20241007 checked |
| P uptake and transport | *pstS* | K02040 | phosphate transport system substrate-binding protein | 438 | Jiang et al. (2023); KEGG 20241007 checked |
| P uptake and transport | TC.PIT | K03306 | inorganic phosphate transporter, PiT family | 360 | KEGG 20241007 phosphate/phosphonate-system annotation |
| P uptake and transport | *phnT* | K11084 | 2-aminoethylphosphonate transport system ATP-binding protein | 5 | Published P-transformation framework; KEGG 20241007 checked |
| P uptake and transport | *phnC* | K02041 | phosphonate transport system ATP-binding protein [EC:7.3.2.2] | 31 | Jiang et al. (2023); KEGG 20241007 checked |
| P uptake and transport | *pstC* | K02037 | phosphate transport system permease protein | 302 | Jiang et al. (2023); KEGG 20241007 checked |
| P uptake and transport | *ugpE* | K05815 | sn-glycerol 3-phosphate transport system permease protein | 60 | Jiang et al. (2023); KEGG 20241007 checked |

Category totals: Inorganic P (Pi) dissolution: 3 KOs/908 gene instances; Organic P (Po) mineralization: 14 KOs/1526 gene instances; P-starvation response regulation: 5 KOs/1514 gene instances; P uptake and transport: 16 KOs/2071 gene instances; total: 38 KOs/6,019 gene instances.

Note: Gene count denotes the number of non-redundant gene sequences assigned to the corresponding KO across the complete metagenomic dataset; it is not a relative-abundance value.

The four functional categories are inorganic P (Pi) dissolution, organic P (Po) mineralization, P-starvation response regulation, and P uptake and transport. KO number and the current KEGG description are reported together to avoid ambiguity arising from reused or historically inconsistent gene symbols.

K00897 (aphA) was retained because the original candidate gene set was predefined according to the published P-cycling framework of Jiang et al. (2023), in which aphA (K00897) was classified as an acid-phosphatase-related gene. In the KEGG 20241007 snapshot used for this project, K00897 is described as kanamycin kinase [EC:2.7.1.95]. This discrepancy is explicitly reported here, and K00897 is not interpreted individually as evidence of organic-P mineralization in the main text.

Reference: Jiang Y, Yang X, Ni K, et al. Nitrogen addition reduces phosphorus availability and induces a shift in soil phosphorus cycling microbial community in a tea (Camellia sinensis L.) plantation. Journal of Environmental Management. 2023;342:118207. doi:10.1016/j.jenvman.2023.118207.

Supplementary Table S6. PERMANOVA and PERMDISP analyses of P-cycling bacterial community composition among fields

| **Taxonomic level** | **Analysis** | **Df** | **Residual df** | **F / pseudo-F** | **R²** | ***p*** |
| --- | --- | --- | --- | --- | --- | --- |
| Phylum | PERMANOVA | 6 | 14 | 136.296 | 0.9832 | 0.001 |
| Phylum | PERMDISP | 6 | 14 | 2.095 | – | 0.127 |
| Genus | PERMANOVA | 6 | 14 | 62.142 | 0.9638 | 0.001 |
| Genus | PERMDISP | 6 | 14 | 10.393 | – | 0.002 |

Note: PERMANOVA and PERMDISP were performed using Bray–Curtis dissimilarities with 999 permutations. PERMANOVA R² values represent the proportion of variation explained by field identity. PERMDISP tests homogeneity of multivariate dispersion among fields; R² is not applicable to PERMDISP.

**Supplementary Figure S1.** Neighbor-Joining phylogenetic tree based on 16S rRNA gene sequences of representative culturable phosphate-solubilizing bacterial isolates from safflower rhizosphere soils. Numbers at nodes indicate bootstrap support values based on 1,000 replicates.
